# RyhB Paralogs Downregulate the Expressions of Multiple Survival-Associated Genes and Attenuate the Survival of *Salmonella* Enteritidis in the Chicken Macrophage HD11

**DOI:** 10.3390/microorganisms11010214

**Published:** 2023-01-15

**Authors:** Xia Meng, Mengping He, Binjie Chen, Pengpeng Xia, Jinqiu Wang, Chunhong Zhu, Heng Wang, Guoqiang Zhu

**Affiliations:** 1 College of Veterinary Medicine, Yangzhou University, Yangzhou 225009, China; 2 International Research Laboratory of Prevention and Control of Important Animal Infectious Diseases and Zoonotic Diseases of Jiangsu Higher Education Institutions, Yangzhou University, Yangzhou 225009, China; 3 Jiangsu Co-Innovation Center for Prevention and Control of Important Animal Infectious Diseases and Zoonoses, Yangzhou University, Yangzhou 225009, China; 4 Department of Animal Husbandry and Veterinary Medicine, Beijing Agricultural Vocational College, Beijing 102442, China; 5 Jiangsu Institute of Poultry Science, Yangzhou 225125, China

**Keywords:** *Salmonella* Enteritidis, RyhB paralogs, *ssaI*, survival, macrophages

## Abstract

RyhB-1 and RyhB-2 are small non-coding RNAs in *Salmonella* that act as regulators of iron homeostasis by sensing the environmental iron concentration. Expressions of RyhB paralogs from *Salmonella* Typhimurium are increased within microphages. RyhB paralogs restrain the growth of *S.* Typhimurium in RAW264.7 macrophages by modulating the expression of *Salmonella* pathogenicity island 1 (SPI-1) genes *sicA* and *rtsB*. However, little is known about the regulatory role of RyhBs and their virulence-associated targets in *Salmonella* Enteritidis. We studied candidate targets of RyhB paralogs via RNA-Seq in conditions of iron limitation and hypoxia. RyhB paralogs were expressed when the *S.* Enteritidis strain CMCC(B)50336 (SE50336) interacted with the chicken macrophage line HD11. We analyzed gene expression associated with *Salmonella* survival and replication in macrophages in wild-type strain SE50336 and the RyhB deletion mutants after co-incubation with HD11 and screened out targets regulated by RyhBs. The expressions of both RyhB-1 and RyhB-2 were increased after co-incubation with HD11 for 8 h and several survival-associated genes within macrophages, such as *ssaI*, *sseA*, *pagC*, *sodC*, *mgtC*, *yaeB*, *pocR*, and *hns*, were upregulated in the *ryhB*-*1* deletion mutant. Specifically, *ssaI*, the type-three secretion system 2 (T3SS-2) effector encoded by SPI-2, which promoted the survival of *Salmonella* in macrophages, was upregulated more than 3-fold in the *ryhB*-*1* deletion mutant. We confirmed that both RyhB-1 and RyhB-2 downregulated the expression of *ssaI* to repress its mRNA translation by directly interacting with its coding sequence (CDS) region via an incomplete complementary base-pairing mechanism. The SPI-2 gene *sseA* was indirectly modulated by RyhB-1. The survival assays in macrophages showed that the ability of intracellular survival of *ryhB*-*1* and/or *ryhB*-*2* deletion mutants in HD11 was higher than that of the wild-type strain. These results indicate that RyhB paralogs downregulate survival-related virulence factors and attenuate the survival of *S*. Enteritidis inside chicken macrophage HD11.

## 1. Introduction

*Salmonella enterica* serovar Enteritidis is a facultative intracellular pathogen that causes nontyphoidal salmonellosis in hosts, such as humans and poultry [1]. As a major foodborne pathogen, *S.* Enteritidis is mainly transmitted through contaminated poultry products and eggs and causes enteritis or food poisoning [2]. When infecting the intestine, *S.* Enteritidis penetrates the mucus layer, invades and internalizes into the intestinal epithelium, and multiplies within non-phagocytic and phagocytic cells. During this process, *S.* Enteritidis must respond to, and cope with, a series of stress conditions in the host internal environment, including iron deficiency and hypoxia [3]. Small non-coding RNA (sRNA) quickly responds to stressful conditions and modulates target gene expression at the post-transcriptional level to resist host defenses [4,5].

Survival and replication of *Salmonella* within macrophages are essential for its pathogenicity in hosts. Many sRNAs contribute to its survival and replication in macrophages [6]. In *Salmonella* Typhimurium, transcriptome analysis showed that 88% of the 280 sRNAs are expressed and 34 sRNAs are upregulated within macrophages, compared to bacterial cultures at the early stationary phase. Among these upregulated sRNAs, RyhB-1 and RyhB-2, two RyhB paralogs, are the most highly upregulated sRNAs [7]. RyhB paralogs regulate a variety of physiological processes in *Salmonella*, including iron homeostasis, nitrate homeostasis, and adaptive response to oxidative stress [8,9,10]. RyhB-1 and RyhB-2 in *Salmonella* Typhi (named as RfrA and RfrB) are essential for the replication of *S.* Typhi inside macrophages [11]. RyhB-1 and RyhB-2 restrict the growth of *S.* Typhimurium within RAW264.7 macrophages by modulating *Salmonella* pathogenicity island 1 (SPI-1) gene expression and directly regulating the mRNAs of the invasion chaperone SicA and the regulatory protein RtsB [6]. Although some targets related to virulence in macrophages have been identified in *S.* Typhimurium, the targets and regulation mechanism of RyhB paralogs in *S.* Enteritidis are poorly understood and require study.

To investigate the regulatory function of *S.* Enteritidis RyhB paralogs during infection of chicken HD11 macrophages, we screened out novel target genes of RyhB paralogs via RNA-Seq and quantitative real-time PCR (qRT-PCR) in vitro and analyzed the regulation mechanism of RyhB paralogs to targets. Furthermore, we analyzed the survival of *S.* Enteritidis mutants lacking RyhB-1 or/and RyhB-2. Our study enriches the regulatory network of RyhB paralogs and provides ideas for reducing *S.* Enteritidis infection.

## 2. Materials and Methods

### 2.1. Bacteria, Plasmids, and Cell-Growth Conditions

The bacterial strains and plasmids used in this study are listed in Table 1. All bacteria were grown in Luria-Bertani (LB) broth or on LB plates at 37 °C with shaking at 180 rpm. Complemented mutants harboring antibiotic-resistance genes were cultured in LB containing ampicillin (Amp, 100 μg/mL) or chloramphenicol (Cm, 34 μg/mL) when appropriate. Anaerobic growth was achieved via static culture at 37 °C in the anaerobic workstation (DG250, Don Whitley Scientific, Bingley, UK) with mixed gas (10% H_2_, 10% CO_2_, and 80% N_2_). For RNA-Seq analysis, all strains were cultured under iron-limited, hypoxic, and nutrient-limited conditions. This stressful culture was achieved by culturing the *S*. Enteritidis wild-type (WT) strain and all the deletion mutants in a medium containing 0.05 mol/L KH_2_PO_4_ and 10 g/L trypsin with 0.2 mM 2, 2′-dipyridyl at 37 °C in an anaerobic workstation [12]. Chicken macrophage HD11 cells (accession number OTWO, HTX2259) were cultivated in Dulbecco’s Minimal Essential Medium (DMEM) (HyClone, Logan, UT, USA) containing 10% heat-inactivated fetal bovine serum (FBS) (Gibco, Carlsbad, CA, USA) and 1% chicken serum (VivaCell, Shanghai, China). Cells were maintained in an atmosphere of 5% CO_2_ at 37 °C.

### 2.2. RNA Isolation, rRNA Removal, Library Preparation, and Sequencing

*S.* Enteritidis WT strain SE50336 and the mutants ∆*ryhB*-*1*, ∆*ryhB*-*2*, and ∆*ryhB*-*1*∆*ryhB*-*2* were grown overnight in 50 mL of the LB medium with 160 rpm shaking at 37 °C under aerobic conditions, washed thrice, and resuspended in an iron-limited and nutrient-limited medium. The above-prepared bacterial cells were cultured for 2 h at 37 °C in an anaerobic workstation. Each sample was analyzed in triplicate. Comparative transcriptomics analyses between the WT strain and the RyhB mutants were performed as follows. Briefly, total RNA of the above stains was extracted using TRIzol reagent (Invitrogen, Waltham, MA, USA) with DNase digestion (Takara, Kusatsu, Japan) according to the manufacturer’s instructions. Ribosomal RNA was removed using a Ribo-Zero rRNA removal kit (Illumina, San Diego, CA, USA) and the mRNA was maintained. Sequencing libraries were prepared using the NEBNext Ultra Directional RNA Library Prep Kit for Illumina (NEB, Ipswich, MA, USA). Then, the library preparations were sequenced on an Illumina Hiseq 2000 platform at Beijing Novogene Bioinformatics Technology Co., Ltd. The RNA-Seq clean reads were aligned to the *S*. Enteritidis str. P125109 genome from NCBI (https://www.ncbi.nlm.nih.gov/nuccore/NC_011294.1, accessed on 10 January 2019) using Bowtie2-2.2.3. Rockhopper was used for RNA-Seq data analysis (https://cs.wellesley.edu/~btjaden/Rockhopper/index.html, accessed on 8 February 2019), including differential gene expression detection, novel and reference-based transcript identification, and operon prediction [13,14]. Gene expression was quantified as reads per kilobase of coding sequence per million reads (RPKM). Genes with adjusted *q* values < 0.01 and (log_2_(fold change)) >1 were assigned as differentially expressed genes (DEGs).

### 2.3. Quantitative Real-Time PCR

Bacteria were grown in LB broth at 37 °C and aerated with a shaker at 180 rpm. They were co-incubated with HD11 cells in a 6-well plate (Corning Inc., Corning, NY, USA) at an MOI of 100:1 (bacteria:HD11 cells) at 37 °C with 5% CO_2_. After infection for 1 h, the cells were washed twice with phosphate-buffered saline (PBS) solution and incubated with DMEM containing 50 µg/mL gentamycin. At 1 and 8 h post-infection (hpi), the medium was removed, cells were washed twice with PBS solution, and then 1 ml TRIzol reagent (Tiangen, Beijing, China) was added to each well for total RNA extraction. The cDNA was synthesized using the HiScript III RT SuperMix (Vazyme, Nanjing, Jiangsu, China). Relative transcript abundance was determined using qRT-PCR with AceQ qPCR SYBR Green Master Mix (low Rox Premixed) (Vazyme). To validate the accuracy of the RNA-Seq results, 13 genes were randomly selected and detected by qRT-PCR. All the primers used are listed in Table S1. Assays were performed in triplicate, and all the data were normalized to the endogenous reference gene *gyrA* using the 2^−∆∆CT^ method.

### 2.4. Prediction of Interactive Sites between RyhB and Target Genes

The prediction of interactive sites between RyhBs and their candidate target genes *ssaI* and *sseA* was carried out as described previously [12,15,16,17]. The whole sequence of *ryhB*-*1*/*ryhB*-*2* and a 162-nt sequence of *ssaI* (12 bases in the whole 5′ untranslated region (5′ UTR) sequence and the first 150 bases in the coding sequence) were submitted to the IntaRNA website to predict the *ssaI-ryhB* interaction site. A 300-nt sequence, including 150 bases in 5′ UTR of *sseA* and 150 bases in coding sequence of *sseA*, was used for the prediction of *sseA-ryhB* interaction site by the same method.

### 2.5. Determination of Interactions between RyhB-1, RyhB-2, and Target Genes via GFP-Based Reporter System

The interaction of sRNA target was detected using the GFP-based fluorescence reporter system, as described previously [12,18]. *E. coli* strain Top10, the plasmids pXG-10SF, *ryhB*-*1*/pJV-300, and *ryhB*-*2*/pJV-300 constructed previously [12] were used in this study. The primers used for the fusion plasmid construction are provided in Table S2. The fusion plasmids *ssaI*-pXG-10SF and *sseA* -pXG-10SF were constructed using the sequence and ligation-independent cloning (SLIC) technology [19]. The interaction between RyhB-1/RyhB-2 and *ssaI* was determined by detecting the fluorescence of *E. coli* TOP10 harboring the *gfp* fusion plasmid and sRNA expression plasmid (named as *ryhB*-*1*::*ssaI*-*gfp* and *ryhB*-*2*::*ssaI*-*gfp* separately) and *E. coli* TOP10 harboring *gfp* fusion plasmid and pJV-300 (named as “no sRNA::*ssaI*-*gfp*”) in both whole-cell colony plates and whole-cell liquid medium. The fluorescence determination on plate and in liquid culture was performed as described previously [12]. In addition, the expression of GFP protein in the above strains was measured by Western blot [12]. The interaction between RyhB-1/RyhB-2 and *sseA* was determined using the same method.

### 2.6. Intra-Macrophage Survival and Replication Assay

The chicken macrophage HD11 cells were cultured in DMEM (HyClone) containing 10% FBS and 1% chicken serum at 37 °C in 5% CO_2_. A monolayer of HD11 cells (1 × 10^6^ per well) was infected with bacteria at a multiplicity of infection (MOI) of 100 at 37 °C in a 6-well plate. After 1 h of infection, the infected cells were gently washed twice with PBS solution and incubated with DMEM containing 50 µg/mL gentamycin. At 1 hpi and 8 hpi, the infected cell monolayers were washed twice with PBS and lysed with 0.5% Triton X-100 (Solarbio, Beijing, China) for 30 min. The lysates were serially diluted, plated on LB agar plates, and cultured overnight at 37 °C to calculate colony forming units (CFUs).

### 2.7. Statistical Analysis

The data from qRT-PCR and intra-macrophage survival assays were analyzed with SPSS 17.0 software (SPSS, Chicago, IL, USA). One-way ANOVA was used for variance analysis. The data from fluorescence measurement was analyzed using an unpaired Student’s *t*-test. The *p*-value ≤ 0.05 was considered statistically significant. Three biological replicates were used in each experiment with three technical replicates.

## 3. Results

### 3.1. Identification of Potential RyhB-1 and RyhB-2 Targets under Iron-Limited and Hypoxic Conditions

We assessed the transcriptomes of various strains (WT, ∆*ryhB*-*1*, ∆*ryhB*-*2*, and ∆*ryhB*-*1*∆*ryhB*-*2*) through RNA-seq technology. The RNA-seq datasets were deposited in GEO (https://www.ncbi.nlm.nih.gov/geo/query/acc.cgi?acc=GSE201112, accessed on 23 April 2022). After a series of quality assessments and sequencing, the clean reads with high quality were obtained by removing contaminated and low-quality sequences. All reads were mapped onto the published reference genome (*S*. Enteritidis str. P125109) by Rockhopper. In this study, more than 95% of the total reads of each sample were successfully aligned to the reference genome (Table S3), which suggested that the clean reads with high quality were credible for further analysis.

**Figure 1:**
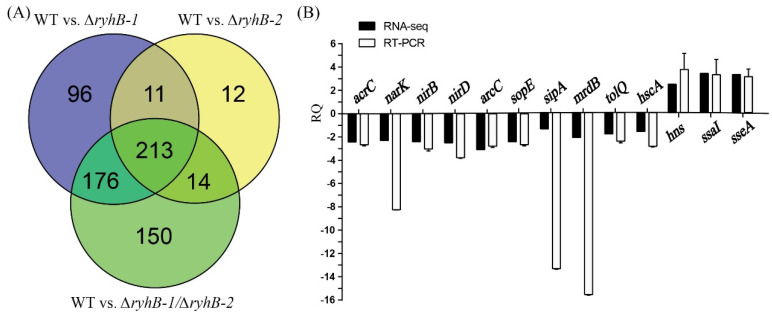
Analysis and verification of differentially expressed genes (DEGs) screened from RNA-seq data. (**A**) Venn diagrams were created using Venny (https://bioinfogp.cnb.csic.es/tools/venny/index.html, accessed on 6 March 2019); (**B**) 13 DEGs that were randomly selected were verified by quantitative real-time PCR (qRT-PCR). RQ means relative quantification values of gene expression in the ∆*ryhB*-*1*∆*ryhB*-*2* mutant relative to that in the wild-type (WT) strain.

The threshold value of significance for DEGs was defined as |log_2_(FC)|≥1 and *q*-value < 0.01. Compared to the WT strain, 496, 250, and 553 DEGs were identified in ∆*ryhB*-*1*, ∆*ryhB*-*2*, and ∆*ryhB*-*1*∆*ryhB*-*2*, respectively. Statistical analysis of the number of individually or commonly regulated genes in the above strains was conducted using a Venn diagram (Figure 1A). The transcriptional level of 213 genes changed in the ∆*ryhB*-*1*, ∆*ryhB*-*2*, and ∆*ryhB*-*1*∆*ryhB*-*2* mutants, which indicated that these genes are common candidate targets of both RyhB-1 and RyhB-2. The transcriptional levels of 176 genes changed in ∆*ryhB*-*1* and ∆*ryhB*-*1*∆*ryhB*-*2* but not in ∆*ryhB*-*2*, which implied that these genes are regulated only by RyhB-1. Similarly, 14 genes were regulated only by RyhB-2. Furthermore, 96 DEGs were only identified in ∆*ryhB*-*1*, and 12 DEGs were only identified in ∆*ryhB*-*2* but not in ∆*ryhB*-*1*∆*ryhB*-*2*. Moreover, 150 DEGs were only identified in ∆*ryhB*-*1*∆*ryhB*-*2* but neither in ∆*ryhB*-*1* nor in ∆*ryhB*-*2*. We inferred that these genes were common targets of both sRNAs, and the transcriptional level of one of the sRNAs increased to regulate these genes when another sRNA was deleted, i.e., one of the sRNAs could complement the regulatory function of another sRNA in these genes.

qRT-PCR was used to validate the RNA-Seq results obtained in this study. We randomly selected three upregulated DEGs (*hns*, *ssaI*, and *sseA*) and 10 downregulated DEGs (*sipA*, *sopE*, *arcC*, *narK*, *nirB*, *nirD*, *narG*, *tolQ*, *hscA,* and *mrdB*) (details in Supplementary Table S4), which were identified in ∆*ryhB*-*1*∆*ryhB*-*2* compared to the WT. These results showed that the relative quantity (RQ) values of the above target genes exhibited similar expression patterns with those data obtained from RNA-Seq, although quantitative differences were observed in three genes (*sipA*, *narK*, and *mrdB*) (Figure 1B). These findings indicated that the RNA-seq data were accurate and, thus, were utilized for subsequent study.

**Figure 2:**
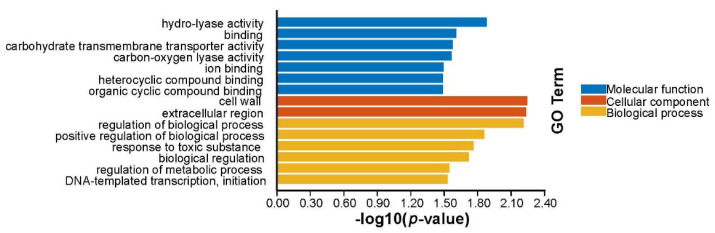
The enriched gene ontology (GO) terms identified from the ∆*ryhB*-*1*∆*ryhB*-*2* mutant compared to the wild-type (WT) strain.

Based on the above Venn diagram analysis, we then focused on the common and individual regulated genes, i.e., 553 DEGs identified in ∆*ryhB*-*1*∆*ryhB*-*2* compared to the WT. Functional annotation of these enriched genes was performed using Gene Ontology and Kyoto Encyclopedia of Genes and Genomes databases. The results revealed that the DEGs were significantly enriched in nine functional terms that included “cofactor transporter activity,” “heme transporter activity,” and “heme-transporting ATPase activity,” which collectively describe transporter activity of the molecular function ontology, and two terms “cofactor transport” and “heme transport” and four terms “protein complex biogenesis,” “protein complex assembly,” “cellular protein complex assembly,” and “cytochrome complex assembly,” respectively, enriched the biological process ontology of localization and cellular component biogenesis (Figure 2). Iron-metabolism-related genes, such as heme transport and cytochrome synthesis, were candidate targets of RyhB-1 and RyhB-2, which were also considered to be the targets of RyhB in *E. coli* [20] and *Shewanella oneidensis*, respectively [21].

KEGG pathway enrichment analysis revealed that 553 DEGs were successfully mapped to 80 different KEGG pathways. We filtered the pathways with *p* value < 0.05 and obtained 14 significantly enriched pathways. According to these significant pathways, we determined that the most significantly enriched pathways by DEGs were the pertussis pathway that consisted of 10 genes (outer-membrane fimbrial usher genes *lpfC*, *stbC*, *bcfC*, *stdB*, *pegC*, *stiC*, *stfC*, *SEN4249*, *SEN2795*, and fimbrial gene *stbA*), followed by the two-component system pathway and signal transduction pathway, each with 27 genes, and the infectious disease pathway, with 13 genes. The Environmental Information Processing pathway also showed the highest DEG enrichment. DEGs also enriched several pathways that were related to the virulence of *S.* Enteritidis, such as bacterial invasion of epithelial cells (SPI-1 type III secretion system guanine nucleotide exchange factor *sopE* and *sopE2*, intimin-like inverse autotransporter *sinH*), and flagellar assembly (*flgD*, *flgI*, *flhB*, *fliE*, *fliF*, *fliI*, *fliJ*).

### 3.2. RyhB-1 and RyhB-2 in SE50336 Are Significantly Increased Inside Chicken Macrophage HD11

**Figure 3:**
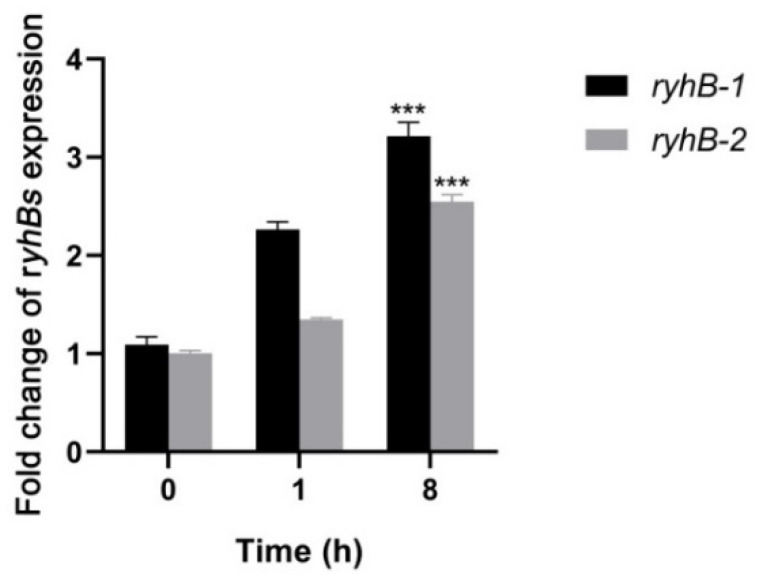
Expressions of RyhB-1 and RyhB-2 from SE50336 infecting HD11 macrophages. Total RNA was extracted at 1 h post-infection (hpi) and 8 hpi, respectively. The relative expressions of RyhB-1 and RyhB-2 were determined using qRT-PCR. The expression values were normalized to the levels of the reference gene *gyrA* and calculated using the 2^−∆∆CT^ method. Values in *y*-axis mean quantification values of RyhB-1/RyhB-2 expression at 1 hpi and 8 hpi relative to that at 0 hpi. All assays were performed in triplicate. *** *p* < 0.001, one-way ANOVA.

RyhB-1 and RyhB-2 of *S.* Typhimurium were significantly induced within murine J774 or RAW264.7 macrophages [7,22]. Both of them restrained *S.* Typhimurium growth inside the macrophages by downregulating SPI-1 gene expression [6]. To investigate the transcription levels and function of RyhB-1 and RyhB-2 in *S.* Enteritidis, the expression of sRNAs in SE50336 was determined by qRT-PCR after co-incubating with HD11 for 1 h or 8 h. The transcription levels of RyhB-1 and RyhB-2 increased by 3.2- and 2.5-fold, respectively, after incubation with HD11 for 8 h compared to that for 0 h (Figure 3). This indicated that the expression of both RyhB-1 and RyhB-2 was increased within macrophages.

### 3.3. RyhB-1 and RyhB-2 from SE50336 Downregulated Survival-Associated Gene Expression during Infection of HD11 Macrophages

**Figure 4:**
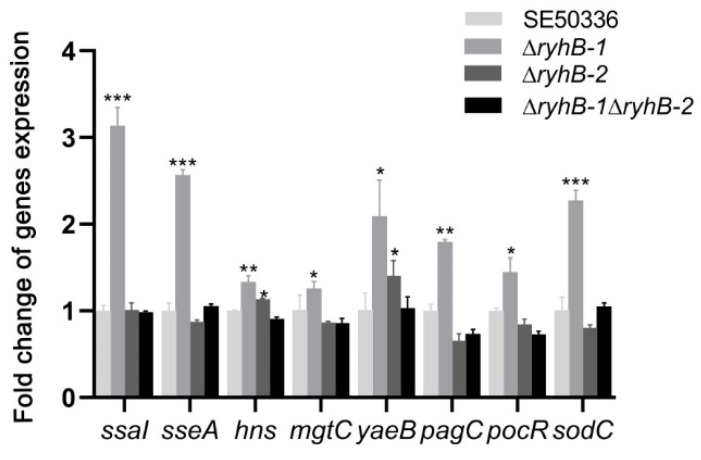
Expressions of survival-associated genes from intracellular bacteria infecting HD11 macrophages. HD11 macrophages were infected with the WT strain SE50336, the mutants ∆*ryhB-1*, ∆*ryhB-2*, and ∆*ryhB-1*∆*ryhB-2*. Total RNA was extracted at 8 hpi and the relative expressions of survival-associated genes were determined using qRT-PCR. The expression values were normalized to the levels of the reference gene *gyrA*. Values on *y*-axis were represented as fold change in gene expression relative to SE50336. All assays were performed in triplicate. * *p* < 0.05, ** *p * < 0.01, *** *p* < 0.001, one-way ANOVA.

Survival and multiplication inside macrophages are important characteristics of *Salmonella* species. It is essential for *Salmonella* to adapt to stress conditions, such as iron deficiency and hypoxia, within macrophages [23]. Our previous data of RNA-Seq showed that the expression of several survival-associated genes changed when *Salmonella* encountered adverse environmental conditions in vitro. To determine if RyhB paralogs affect the survival ability of *Salmonella* in macrophages, we studied the expression levels of survival-related genes in *ryhB* deletion mutants (∆*ryhB*-*1*, ∆*ryhB*-*2,* and ∆*ryhB-1*∆*ryhB-2*) and compared them with the WT strain SE50336. The genes included *ssaI*, *sseA*, *pagC*, *sodC*, *mgtC*, *yaeB*, *pocR*, and *hns*. All of these genes were upregulated in the ∆*ryhB-1* mutant after infection of HD11 for 8 h. In particular, *ssaI*, the type-three secretion system 2 (T3SS-2) effector encoded by SPI-2, which promotes the survival of *Salmonella* in macrophages, was upregulated more than 3-fold in the ∆*ryhB-1* mutant compared to the WT strain (Figure 4). The expression of *sseA*, a chaperone in SPI-2, was also upregulated in the ∆*ryhB-1* mutant. Except for *hns* (encoding a histone-like nucleoid structuring protein) and *yaeB* (encoding an RNA methyltransferase), the expression levels of all other genes in the ∆*ryhB-2* mutant were not significantly changed compared to levels in the WT strain. Expression levels of the eight survival-associated genes were not changed in the ∆*ryhB-1*∆*ryhB-2* mutant. These results indicated that RyhB-1 plays an important role in regulating the expression of target genes within macrophages.

### 3.4. RyhB Paralogs Directly Interact with the SsaI mRNA

**Figure 5:**
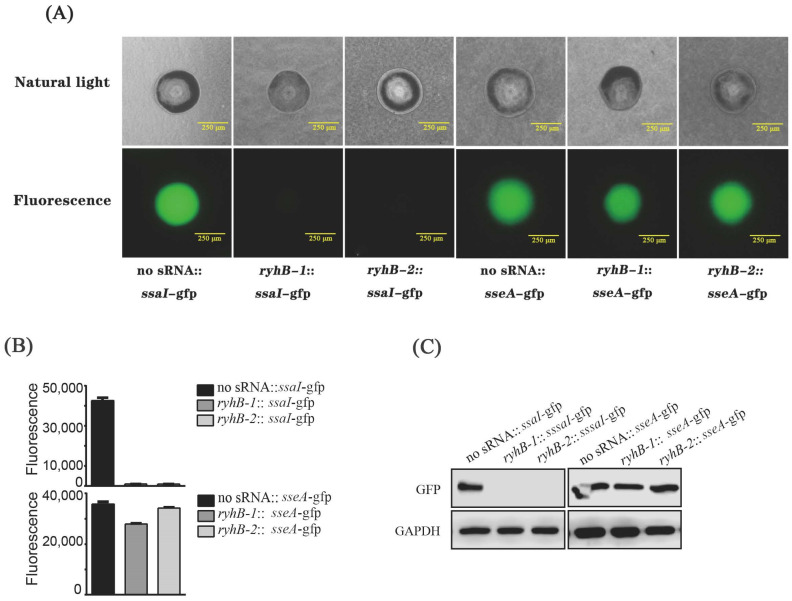
Regulation of *ssaI* and *sseA* by RyhB paralogs. (**A**) Single colonies on Luria-Bertani (LB) plates imaged using natural-light mode and fluorescence mode, respectively, by an inverted fluorescence microscope. (**B**) Fluorescence units of bacteria when cultured in LB liquid medium. The data were analyzed statistically using an unpaired Student’s *t*-test. (**C**) The expression of GFP protein in *E. coli* TOP10 containing various fusion plasmids was determined by Western blot. GAPDH was used as a loading control.

The candidate targets of RyhB-1 and RyhB-2 were predicted by the IntaRNA program. The predicted result of the RyhB-*ssaI* interaction site suggested that a region (nt 15–23) in RyhB-1 could form base pairs with the coding sequence (CDS) region (nt 188–196 in a 297-nucleotide sequence) of *ssaI* (Figure S1A), while a region (nt 35–43) in RyhB-2 could form base pairs with the CDS region (nt 219–227 in a 297-nucleotide sequence) of *ssaI* (Figure S1A). RyhB-1was predicted to interact with the 5′ UTR (nt 86–94) of *sseA*, while RyhB-2 was predicted to interact with a similar region in 5′ UTR (nt 78–94) of *sseA* (Figure S1B). We used a GFP-based reporter system to investigate the interaction between RyhB homologs and targets. The fluorescence intensity of the strains carrying *ryhB-1*::*ssaI*-gfp or *ryhB-2*::*ssaI*-gfp was visually weaker than the strain with the “no sRNA::*ssaI* -gfp” on LB agar plates (Figure 5A). Liquid cultures of the above strains were measured for whole-cell fluorescence when the OD_600_ of cell density was 2. The fluorescence units of the strains harboring *ryhB-1*::*ssaI*-gfp and *ryhB-2*::*ssaI*-gfp were 42.6-fold and 43.8-fold lower than that of “no sRNA::*ssaI*-gfp”, respectively (Figure 5B). Detection of GFP protein expression also revealed that the strains containing plasmid *ryhB-1*::*ssaI*-gfp or *ryhB-2*::*ssaI*-gfp expressed less GFP protein than the “no sRNA::*ssaI*-gfp”, which is consistent with the results of liquid culture whole-cell fluorescence measurement (Figure 5C). These data indicate that RyhB-1 and RyhB-2 interact with the CDS region of *ssaI* and affect the expression of SsaI-GFP fusion protein. These findings suggest that RyhB-1 and RyhB-2 directly downregulate the expression of SsaI protein by interacting with the CDS region of *ssaI*. The interaction between RyhB paralogs and *sseA* was studied using this GFP reporter system. Although the interaction site prediction showed that RyhB paralogs may interact with *sseA*, the results of both fluorescence measurement and GFP protein expression demonstrated that RyhB-2 did not affect the expression of GFP, while RyhB-1 slightly downregulated the expression of GFP (Figure 5).

### 3.5. RyhB Paralogs Decrease the Survival Capability of S. Enteritidis Inside HD11 Macrophages

**Figure 6:**
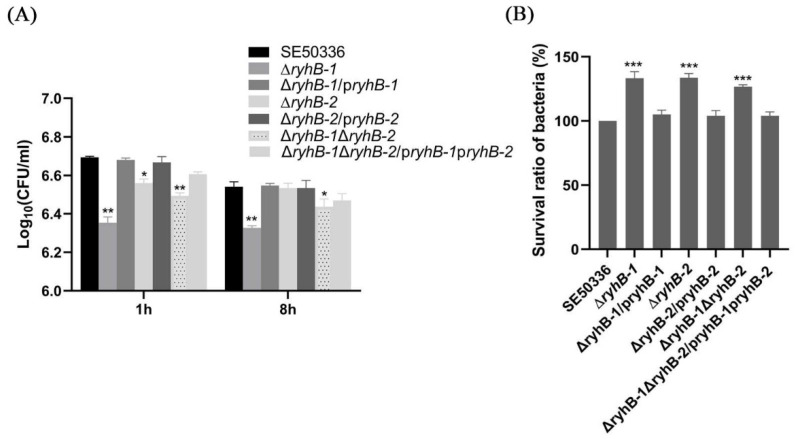
Effects of RyhB paralogs on the survival of *S.* Enteritidis within HD11 macrophages. (**A**) The number of intracellular bacteria was determined at 1 hpi and 8 hpi by plating serial dilutions of HD11 lysates on LB plates for counting CFU. The comparison of intracellular bacteria numbers between the mutants and the WT strain was analyzed statistically using one-way ANOVA. * *p* < 0.05, ** *p* < 0.01. (**B**). Survival ratios of the WT strain and RyhB mutants within HD11 macrophages. The survival ratios of bacteria were calculated in terms of the percentage of bacteria number at 8 hpi to the number at 1 hpi for each strain. To compare with the mutants, the survival ratio of the WT strain was set at 100%. The comparison of survival ratios between the mutants and the WT strain was analyzed statistically using one-way ANOVA. *** *p* < 0.001. All the above assays were performed in triplicate.

To verify the regulatory functions of RyhB-1 and RyhB-2 in the process of infecting macrophages, the WT, deletion mutants (∆*ryhB-1*, ∆*ryhB-2*, and ∆*ryhB-1*∆*ryhB-2*), and complemented mutants (∆*ryhB-1*/p*ryhB-1*, ∆*ryhB-2*/p*ryhB-2*, and ∆*ryhB-1*∆*ryhB-2*/p*ryhB-1*p*ryhB-2*) were used to infect chicken HD11 macrophages for the survival assay. These assays were conducted by counting CFUs at 1 and 8 h post-infection (hpi) with a gentamicin treatment. Compared to the WT, the bacterial number of all three deletion mutants, especially ∆*ryhB-1*, which entered HD11 macrophages for 1 h, was clearly reduced. This revealed that deletion of RyhB-1 and/or RyhB-2 attenuated the invasion ability of macrophages (Figure 6A). By comparing the CFU between 1 h and 8 h post-infection, all the deletion mutants had a significantly higher survival and proliferation than the WT within macrophages (Figure 6B, *p* < 0.001). All of the complemented mutants restored the above phenotypes. These data indicated that deletion of *ryhB-1* or *ryhB-2* increased the survival of *S.* Enteritidis, and the effect of the ∆*ryhB-1* mutant was the greatest. RyhB-1 and RyhB-2, individually or together, contributed to attenuating the intracellular *Salmonella* survival ability in HD11.

## 4. Discussion

*Salmonella* can survive, adapt, and replicate in the stressful environment within macrophages. sRNAs are post-transcriptional regulators that can sense environmental stress signals and can be increased to help the bacteria adapt to stress conditions [4,5]. We demonstrated that the expression of RyhB-1 and RyhB-2 was increased after *S.* Enteritidis infected chicken HD11 macrophages for 8 h. Padalon-Brauch et al. (2008) demonstrated that the transcriptional levels of RyhB-1 and RyhB-2(IsrE) from *S.* Typhimurium increased within murine J773 macrophages at 8 h post-infection, compared with levels in cell culture medium grown for 1.5 h [22]. The intra-macrophage (RAW264.7) transcriptome of *S.* Typhimurium also showed that RyhB-1 and RyhB-2 were upregulated within macrophages compared to *S.* Typhimurium cultures at the early stationary phase. They were the most highly upregulated sRNAs within the macrophages [7]. Although the expression levels of the two RyhB paralogs in *S.* Enteritidis and *S.* Typhimurium were different, the upregulation trend was the same. This indicates that there is a close relationship between RyhB and *Salmonella* survival in macrophages. RyhB paralogs may be important for the survival and replication of *S.* Enteritidis inside macrophages.

Within macrophages, *Salmonella* cells encounter several stress conditions, including iron deficiency, oxidative stress, and nutrient limitation [23]. RyhB-1 and RyhB-2 are iron-regulated sRNA homologs [24]. We previously showed that the expression levels of RyhB-1 and RyhB-2 in *S.* Enteritidis were increased under iron-limited and/or anaerobic conditions [12]. In particular, the expression of RyhB-1 was higher than RyhB-2. RyhB-1 may play a major role in the response to iron deficiency and hypoxia. The expression pattern of RyhB paralogs in iron-limited and/or anaerobic conditions in this study was consistent when *S.* Enteritidis infected macrophages. It is possible that both the intra-macrophage environment and the stressful iron-limited and hypoxic conditions can induce the expression of RyhBs. The stressful conditions of iron deficiency and hypoxia probably reflect the stress of *Salmonella* in macrophages. Therefore, the transcriptomic profile analysis of *S.* Enteritidis under the conditions of iron deficiency and hypoxia may provide a reference for the *Salmonella* transcriptomic profile during the process of macrophage infection. We used RNA-Seq to analyze the transcriptome changes in *S.* Enteritidis caused by deletion of RyhB-1 and/or RyhB-2 under conditions of iron deficiency and hypoxia. Multiple iron-metabolism-related DEGs and virulence-related DEGs were screened out. RyhB is an important regulator of iron homeostasis regulation in bacteria [8]. Our RNA-Seq data showed that the expressions of multiple iron-metabolism-related genes, such as ferredoxin-like genes *fixX* and *ydiT*, and heme exporter genes *ccmB* and *ccmD* were decreased in the RyhBs deletion mutant. This indicated that RyhBs in *Salmonella* upregulated redox of iron. KEGG pathway enrichment analysis showed that the pertussis pathway was mostly enriched. This pathway contains several fimbrial usher protein-encoding genes, such as *lpfC*, *pegC*, and *stfC*. In addition to fimbrial genes that are enriched in the pertussis pathway, other fimbrial genes (e.g., major fimbrial subunit *stfA* and *stdA*, fimbrial chaperone genes *stbB* and *pegB*, curli assembly gene *csgF*) also displayed changes in expression in the *ryhBs* deletion mutant. This indicated that RyhB-1 and RyhB-2 could regulate the formation of fimbriae. In addition, three survival-associated genes within macrophages (*ssaI*, *sseA*, and *hns*) were identified. The expressions of these three genes significantly increased in the mutants ∆*ryhB-1*, ∆*ryhB-2*, and ∆*ryhB-1*/∆*ryhB-2*. To certify whether these three genes were the targets regulated by RyhB paralogs inside macrophages, we used qRT-PCR to detect the expression levels of several survival- and replication-related genes, including *ssaI*, *sseA*, *hns*, *pagC*, *sodC*, *mgtC*, *yaeB*, and *pocR*. In these genes, SsaI and SseA are components in T3SS-2, encoded by SPI-2. T3SS-2 is closely related to the pathogenicity of *Salmonella* [25]. It contains structure proteins and effectors that are required for intracellular survival and replication inside macrophages [26]. SsaI is an early substrate of T3SS that co-regulates SseB secretion with SsaG [27]. SseA is a chaperone for the SseB and SseD translocon components of T3SS, which is a critical virulence factor of *S.* Typhimurium [28,29]. SPI2 is critical for *Salmonella* virulence and proliferation in macrophages [30]. Histone-like nucleoid structuring protein (H-NS) belongs to a family of small nucleoid-associated proteins [31]. It is a global regulator involved in controlling gene expression during the infection cycle of *Salmonella* [32]. H-NS also negatively regulates the expression of invasion-associated genes [33]. The *pagC*, *sodC*, *mgtC*, *yaeB*, and *pocR* genes are also required for survival and replication in macrophages [34,35,36]. Our qRT-PCR results showed that the expression levels of all above genes, especially *ssaI*, *sseA*, and *sodC*, were upregulated in the RyhB-1 mutant, compared to levels in the WT strain. RyhB-1 may play a more important role in the regulation of target genes. RyhB-2 had little effect on the expression of the above genes, except for *hns*. It is unlikely that the deletion of RyhB-1 leads to the upregulation of target genes, while the double deletion of both RyhB-1 and RyhB-2 does not affect target gene expression. The double deletion of RyhBs may cause the compensation of other sRNA or regulatory factors in the regulatory network of *Salmonella*. It is also possible that the RyhB-1 or RyhB-2 binds distinct targets, leading to cancelling each other out in the regulation of those genes.

Although the data of RNA-Seq and qRT-PCR showed that the levels of numerous gene expressions changed after the deletion of RyhB-1 and/or RyhB-2, these genes may not necessarily be directly regulated by RyhB paralogs. Many genes may be indirectly regulated. Although we screened out two candidate target genes (*ssaI* and *sseA*) by a bioinformatics prediction method IntaRNA program, the genes that are not predicted as direct targets are not necessarily the direct targets of RyhB regulation. Our study of the GFP-based reporter system showed that only *ssaI* was a target gene of RyhB-1 and RyhB-2, but *sseA* was not directly regulated by RyhB-1 and RyhB-2. These data show that the bioinformatics prediction method has limitations. The screening of target genes regulated by RyhBs requires additional study. We considered the regulatory mechanism of RyhB-1 and RyhB-2 on *ssaI*. Although RyhB-1 and RyhB-2 share the same 33-bp homologous region and Fur-binding sites, there are differences in the regulation of target genes. The regulatory region of RyhBs to *ssaI* is not in the conserved region of 33 bp, and the regions of *ssaI* that bind to RyhBs are also different. Previous studies showed that RyhB commonly negatively regulates targets by blocking the ribosome-binding site (RBS) and inhibits translation through incomplete complementary base pairing with the 5′-UTR of the target gene. For example, RyhB-1 and/or RyhB-2 can directly bind to the 5’-UTR region of the invasion-related gene *sipA*. This opens the hidden RBS of the secondary structure and promotes the expression of *sipA* [12]. In this study, the predicted RNA-RNA interaction showed a 7 bp base pairing between the CDS region of *ssaI* and RyhBs. Although no other sRNA has been reported to have such short base pairing in the CDS region, the effective interaction region, which is less than 7bp, has been reported in the MicC–*ompD* interaction. MicC targets the *ompD* mRNA by forming a ≤ 12-bp RNA duplex within the CDS and accelerates RNase E-dependent decay of *ompD* mRNA. Importantly, codons 23-26 of *ompD* CDS are essential and sufficient for interaction with MicC [37]. In addition, sRNAs, such as RybB, SdsR, and DicF, have also been reported to bind within the CDS of the target transcript. The sRNA RybB in *S*. Typhimurium represses *ompN* mRNA translational initiation by forming an approximate 16-bp RNA duplex with the CDS region down to the fifth codon [38]. A conserved RpoS-dependent sRNA SdsR represses *ompD* mRNA via binding to the coding sequence of the 15th to 26th codons [39]. DicF interacts with the transcriptional activator *pchA* mRNA CDS directly and specifically to promote PchA expression [40]. Although we predicted the binding of RyhB and *ssaI* CDS, the mechanism of RyhB regulation needs further experimental confirmation.

The assay of survival and replication within macrophages demonstrated that the deletion of RyhB-1 and/or RyhB-2 increased the survival of *S.* Enteritidis inside macrophages. This result is consistent with a previous study showing that RyhB homologs from *S.* Typhimurium reduced the growth within macrophages [6]. However, different target genes regulated by RyhB homologs were identified from *S.* Enteritidis and *S.* Typhimurium. RyhB homologs from *S.* Typhimurium downregulated the expression of SPI-1 genes, such as *rtsB* and *sicA*. However, RyhB-1 from *S.* Enteritidis negatively modulated the expression of SPI-2 genes, *ssaI*, and other survival-associated genes, including *sseA* and *yaeB*. Although *S.* Enteritidis and *S.* Typhimurium belong to the same subspecies, and the sequences of RyhB homologs in *S.* Enteritidis have 100% homology with those in *S.* Typhimurium [12], they may still differ in the regulatory mechanisms of target genes. These differences indicate that there is a significant difference in the regulatory mechanism of RyhBs between the two serovars. In *S.* Enteritidis or *S.* Typhimurium, the RyhB targets and regulation mechanisms remain uncertain and require more research.

When *Salmonella* enters into macrophages, it survives and multiplies by secreting effector proteins and generating the *Salmonella*-containing vacuole (SCV). The SCV helps *Salmonella* develop a systemic infection and form persister cells [3,41]. However, restrained intra-macrophage proliferation limits antigen presentation and development of a rapid CD8 + T-cell response, which contributes to immune evasion and enhances virulence [42,43]. Eriksson et al. confirmed that the virulence of *S.* Typhimurium mutants with overgrowth phenotypes inside macrophages was attenuated [44]. In this sense, restriction of *S.* Enteritidis survival within macrophages mediated by RyhB-1 and/or RyhB-2 is conducive to *Salmonella* virulence. We previously showed that RyhB-1 and/or RyhB-2 could promote adhesion and invasion ability to intestinal epithelial cells by upregulating the expression of the SPI-1 genes *sipA* and *sopE* [12]. In this study, we demonstrated that RyhB-1 and/or RyhB-2 contribute to restraining the survival and replication of *S.* Enteritidis inside macrophages. Invasion into intestinal epithelial cells and survival within macrophages are important pathogenic processes in *Salmonella* infection. In addition, our previous study showed that, compared to the WT strain SE50336, the LD_50_ to the chicken was significantly increased and the colonization of bacteria in the heart, liver, spleen, and lung was decreased when challenged with a *ryhB-1* or/and *ryhB-2* deletion mutant. We demonstrated that the deletion of *ryhB-1* and/or *ryhB-2* attenuated the pathogenicity of *S.* Enteritidis to a 1-day-old chicken [45]. Thus, we conclude that both RyhB-1 and RyhB-2 regulate the pathogenicity of *S.* Enteritidis by modulating several virulence factors, including the SPI-1 and SPI-2 genes.

## Figures and Tables

**Table 1:** Bacteria and plasmids used in this study.

**Strain/Plasmids**	**Characteristics**	**References**
Strains		
CMCC(B)50336	*Salmonella enterica* serovar Enteritidis wild-type	NICPBP, China
∆*ryhB-1*	*ryhB-1* deficient mutant	[12]
∆*ryhB-2*	*ryhB-2* deficient mutant	[12]
∆*ryhB-1*∆*ryhB-2*	*ryhB-1* and *ryhB-2* deficient mutant	[12]
∆*ryhB-1*/p*ryhB-1*	∆*ryhB-1* carrying pBR-*ryhB-1* (Amp^r^)	[12]
∆*ryhB-2*/p*ryhB-2*	∆*ryhB-2* carrying pACYC-*ryhB-2* (Cm^r^)	[12]
∆*ryhB-1*∆*ryhB-2*/p*ryhB-1 ryhB-2*	∆*ryhB-1*∆*ryhB-2* carrying pBR-*ryhB-1* and pACYC-*ryhB-2* (Amp^r^ and Cm^r^)	[12]
Plasmids		
pJV-300	Amp^r^; sRNA cloning vector	[12]
*ryhB-1*/pJV-300	Amp^r^; recombinant vector	[12]
*ryhB-2*/ pJV-300	Amp^r^; recombinant vector	[12]
pXG-10SF	Cm^r^; target gene cloning vector with GFP	[12]

## Data Availability

The datasets used and analyzed during the current study are available from the corresponding author on reasonable request.

## Supplementary Materials

The following supporting information can be downloaded at: https://www.mdpi.com/article/10.3390/microorganisms11010214/s1, Table S1: Primers of genes used for qRT-PCR. Table S2. Primers for construction of recombinant plasmids. Table S3. Summary about total reads successfully aligned to reference genome. Table S4. Information of 13 candidate target genes screened byRNA-Seq. Figure S1: Interaction site prediction between RyhB-1/RyhB-2 and putative target genes.
