# Supplementary material for: RyhB Paralogs Downregulate the Expressions of Multiple Survival-Associated Genes and Attenuate the Survival of Salmonella Enteritidis in the Chicken Macrophage HD11
Source: Microorganisms. 2023 Jan 15;11(1):214. doi: 10.3390/microorganisms11010214 (PMC9860832; doi:10.3390/microorganisms11010214)
Supplement: Supplementary file 1 [file microorganisms-11-00214-s001.zip › microorganisms-2134533-supplementary.pdf]

Supplementary Materials

Table S1. Primers of genes used for qRT-PCR.

Primer name	Sequence (5′–3′)
ryhB-1-F	AAAAAGAAGTAGACAAGTGCGAATG
ryhB-1-R	AGCAATGTGAGCAATGTCGTG
ryhB-2-F	GCGGCTGAAAAAGACCATGA
ryhB-2-R	CCTGTTCGGTACGACATTGCT
ssaI-F	AACCGAGCCAGGAGCAAATTA
ssaI-R	TTATTAAGCGCTTGCGATGCT
sseA-F	GGCAGAGAGCCCCAAAAAGA
sseA-R	CCGGGGCTTGAGCATTAAGT
pagC-F	TATCCGAGGGGTAAATGTGA
pagC-R	CAGCGCGTATAACGAAAAAT
sodC-F	GTAGAGATGAATCTGGTGACG
sodC-R	TTCATGCTTGCCGGTATTT
mgtC-F	TGTCTCTGGTATTGGCTTTC
mgtC-R	CGGGAGTTGATTTATACGCT
yaeB-F	GAAATTAGGCAGTCTGGATCT
yaeB-R	CGTTAAAATCATGCAGCCAG
pocR-F	GCCTCACATGTCTATCTCAG
pocR-R	AGACTTTGCAAAAATAGCTGG
hns-F	TTCTGAACAACATCCGTACT
hns-R	CTATTCAGCAGTTCATTCGG
arcC-Fwd	ATTGCTGGCGGAGCAGATAG
arcC-Rev	CCGCTTCACATACCCGCTTA
nark-Fwd	GGTATCCTCATCGTGCCCTG
nark-Rev	TATTGCCCAGACCACCGTTC
nirB-Fwd	ATCATCGCCACGGAGAAAGG
nirB-Rev	GGGTCAGTTTGTCGGCAGTA
nirD-Fwd	CGCGATTTTCCGTCCTTACC
nirD-Rev	CGCAATCAGTCCACGAGACA
narG-Fwd	ACCAACGTTCACGAGCTGAT
narG-Rev	AGGAAGTTGAGCGCCTTCTC
sipA-Fwd	AGGCGAAGACGCTACTGATG
sipA-Rev	GCTTCGCTTCCGCTTTCTTT
sopE-Fwd	AGGTAGCGCGAGTAAAGACC
sopE-Rev	CGTCATTCTTGGTTGCTCCG
tolQ-Fwd	CGGGTATTGCCGAAGCATTG
tolQ-Rev	TACCCCTTGTTGCTTTCGCT
hscA-Fwd	CGCTATCCGCATCTGCCTTA
hscA-Rev	CGAAATAGGCGGGAACGGTA
nrdB-Fwd	TCTGCTGGCGTTACTGGTTT
nrdB-Rev	CATCACCACCATGACCACCA

Table S2. Primers for construction of recombinant plasmids.

Primer name	Sequence (5′-3′)
ssaI-pXG-10SF-Fwd	GAGATACTGAGCACATGCATAATATTTCTGATATGAGCGTAGTGC
ssaI-pXG-10SF-Rev	AGCCAGCGGATCCGCTAGCTGCCATGTTATCCAGCTT
sseA-pXG-10SF-Fwd	GAGATACTGAGCACATGCATTTTCATCCCTGGCCTTAACG
sseA-pXG-10SF-Rev	AGCCAGCGGATCCGCTAGCCCTTTTTGTTTTTCCTGACGG
pXG-10SF-seq-Fwd	CGCCAGATATCGACGTCT
pXG-10SF-seq-Rev	AAAGACAGGCACCTCTCC

Table S3. Summary about total reads successfully aligned to reference genome.

Sample name	Total reads	Successfully aligned reads	Ratio
WT1	4474322	4305857	96%
WT2	5471969	5207970	95%
WT3	5579891	5293677	95%
Δ*ryhB-1-1*	5476167	5256237	96%
Δ*ryhB-1-2*	5814310	5544776	95%
Δ*ryhB-1-3*	5756354	5526504	96%
Δ*ryhB-2-1*	5969797	5712121	96%
Δ*ryhB-2-2*	6090677	5817934	96%
Δ*ryhB-2-3*	5725666	57554	96%
Δ*ryhB-1*Δ*ryhB-2-1*	5767623	5505745	95%
Δ*ryhB-1*Δ*ryhB-2-2*	6323152	6000992	95%
Δ*ryhB-1*Δ*ryhB-2-3*	6007256	5713601	95%

Table S4. Information of 13 candidate target genes screened byRNA-Seq.

Gene name	Product	log_2(FC)		
		WT vs. Δ*ryhB-1*	WT vs. Δ*ryhB-2*	WT vs. Δ*ryhB-1*/Δ*ryhB-2*
sipA	pathogenicity island 1 effector protein	−1.043961057	−0.449383288	−0.492440637
sopE	guanine nucleotide exchange factor SopE	−1.30455987	NS	−1.284453795
hns	global DNA-binding transcriptional dual regulator H-NS	1.314465854	1.825069442	1.278914736
ssaI	pathogenicity island protein	2.40329259	1.563195229	1.749635333
sseA	pathogenicity island protein	1.749390717	1.269857594	1.708645919
arcC	carbamate kinase	NS	NS	−1.280819577
narK	NarK family nitrate/nitrite MFS transporter	−1.372546441	−1.255806586	−1.62991254
nirB	nitrite reductase large subunit	−1.269562961	−1.118081493	−1.325694806
nirD	nitrite reductase small subunit NirD	NS	NS	−1.268219173
narG	carbamate kinase	NS	NS	−1.204395098
tolQ	colicin uptake protein TolQ	0.355563326	0.33111866	0.541312818
hscA	chaperone protein HscA	0.498653077	0.712040375	0.614042734
mrdB	cell wall shape-determining protein	0.664299108	0.765219803	0.466340675

WT: wild-type; NS: not significant.

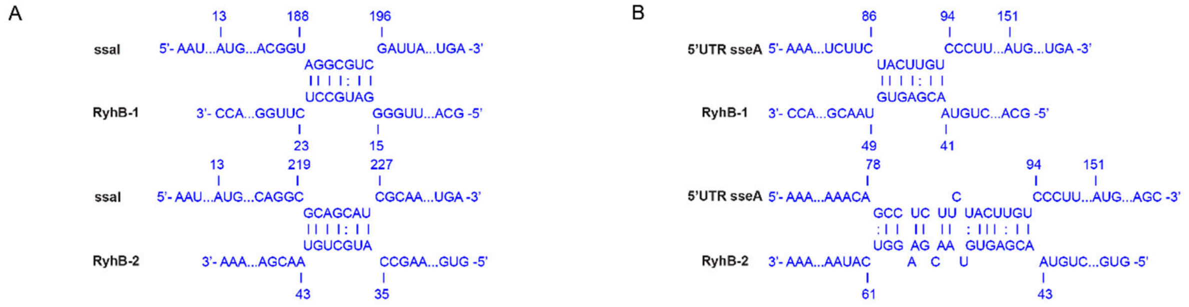

Figure S1. Interaction site prediction between RyhB-1/RyhB-2 and putative target genes. (**A**) Prediction between RyhB-1/RyhB-2 and *ssaI*. CDS region of *ssaI* is predicted to form base pairs with RyhB-1 and RyhB-2. (**B**) Prediction between RyhB-1/RyhB-2 and *sseA*. 5′ UTR of *ssaI* is predicted to form base pairs with RyhB-1 and RyhB-2.
